# Obstructive Jaundice in an Elderly Female

**DOI:** 10.4103/1319-3767.70636

**Published:** 2010-10

**Authors:** Nisar A. Wani, Tasleem L. Kosar, Aijaz A. Rawa

**Affiliations:** Deptartment of Radiodiagnosis and Imaging, Sher-I- Kashmir Institute of Medical Sciences, Srinagar, Jammu & Kashmir, India

A 55-year-old woman presented with 1 month history of right hypochondrium pain, vomiting, and jaundice. On examination, she had tender right hypochondrium and palpable gall bladder. Blood investigations revealed increased white cell count with eosinophilia; serum bilirubin (6 mg/dL) and alkaline phosphatase (900U/L) were raised; alanine aminotransferase (ALT) and aspartate aminotransferase (AST) were not significantly elevated. Ultrasonography showed a cyst in the right lobe of liver and dilated common bile duct (CBD) filled with echogenic debris. Gall bladder was distended and transonic. MRCP was performed for further evaluation. Cholangiographic thick slab MRCP revealed a cyst in the right lobe of liver closely related to right lobe ducts, with intracystic linear and irregular hypointensities; intrahepatic bile ducts were dilated and CBD filled with long, low signal intensity mass like structure. GB was distended and pancreatic duct slightly dilated [Figure 1].

**Figure 1:**
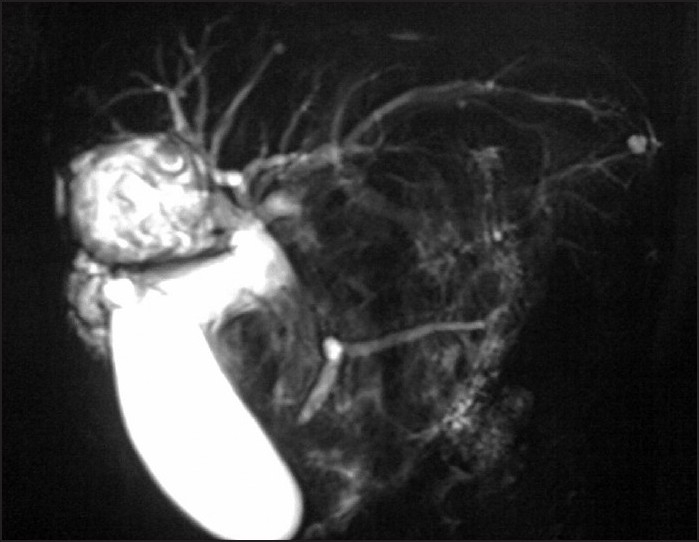
Thick slab coronal plane MRCP image showing dilated intrahepatic bile ducts and a cyst closely related to right lobe ducts with equivocal evidence of communication. Cyst shows internal low signal intensity membranes; common bile duct is filled with long, linear mass like hypointense signal intensity structure. Gall bladder is distended, and pancreatic duct is slightly dilated

## QUESTION

What is the diagnosis?

## ANSWERS

**Figure 2:**
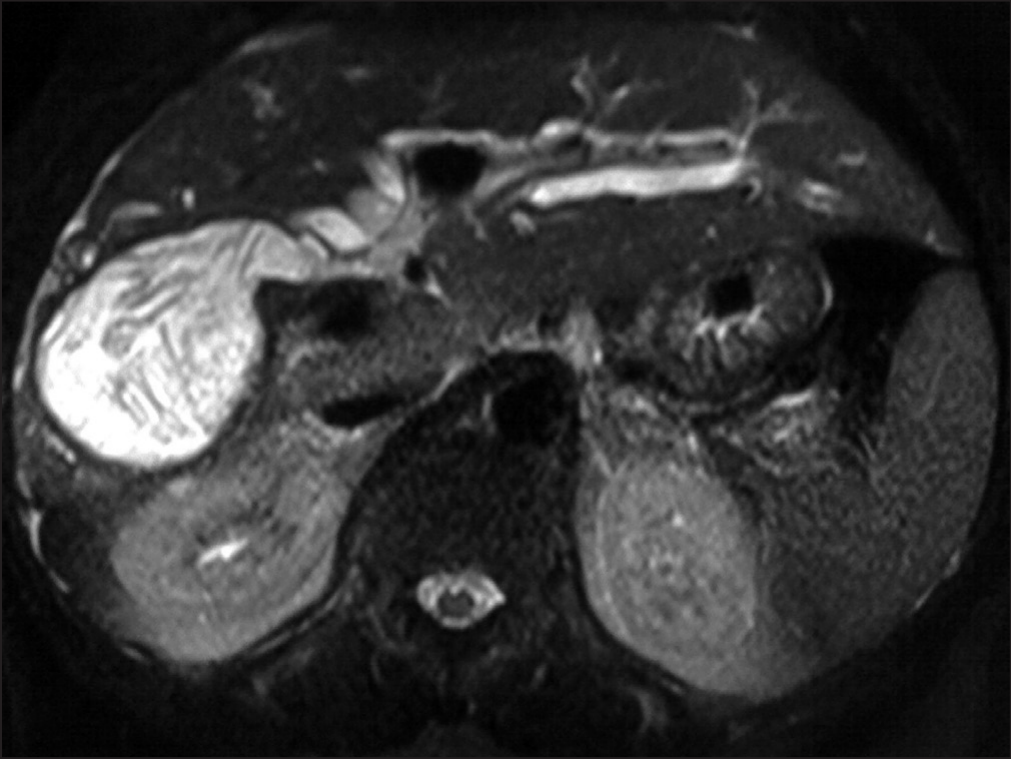
Axial T2W MR image showing a cyst in the right lobe of liver posteriorly; medial aspect of the cyst shows a beak like projection from the wall into the right posterior sectoral duct. Membranes are seen within the cyst

**Figure 3:**
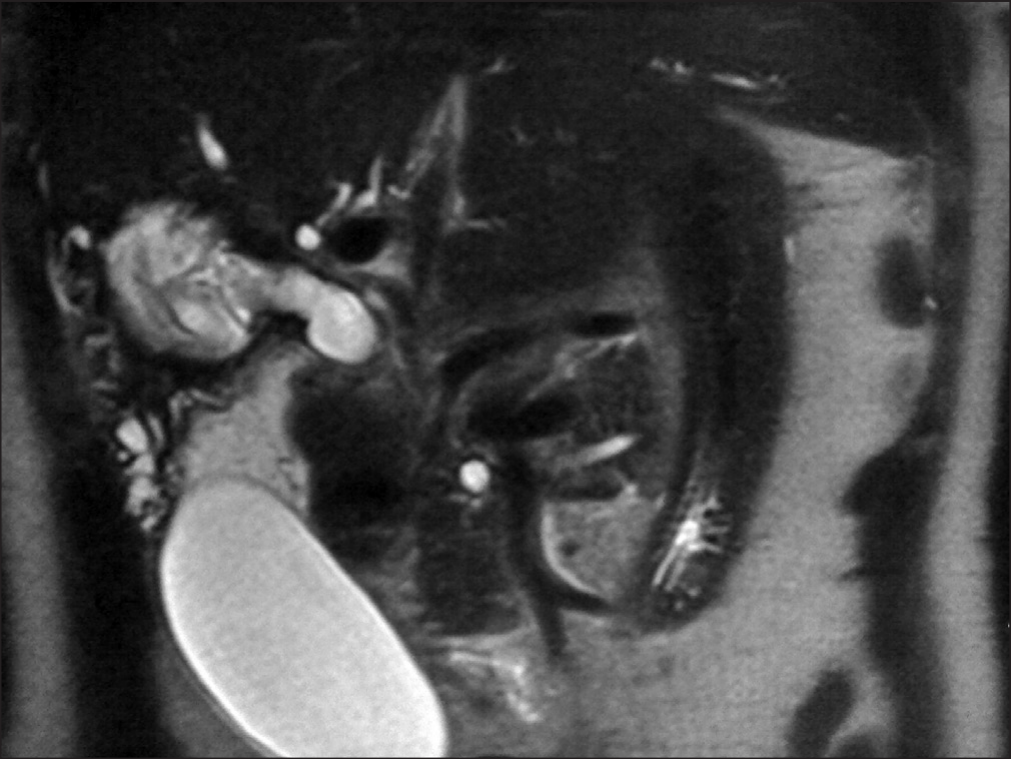
Coronal T2W MR image shows a definite communication of the liver cyst with the right posterior sectoral duct

Transverse and coronal plane heavily T2 weighted, thin slice MR images revealed the diagnosis. T2W axial and coronal MR images depicted a cyst in the right lobe with a focal beak like projection from the medial wall of the cyst extending toward and communicating with the right posterior sectoral duct; the cyst had a smooth thin wall and internal membranes [Figures 2 and 3]. These findings were consistent with cystobiliary communication. MRCP findings were highly suggestive of hepatic hydatid cyst rupture into the biliary tract. ERCP was performed and membranes removed from the common duct and the cyst cavity was drained. The patient improved symptomatically and her LFT returned to normal.

Intrabiliary rupture of hydatid cyst is an imaging diagnosis. Following imaging findings have been described as suggestive of rupture of hydatid cyst into the biliary tract.[1,2]

1. Cyst in the liver showing some irregularity of the wall, and hydatid sand, membranes and scolices inside.
2. Communication between the liver cyst and dilated bile duct proximal to porta hepatis seen as focal discontinuity of the cyst wall.
3. Dilated bile duct containing ruptured cyst membranes.

Low signal intensity contents within the dilated CBD on MRCP may be due to sludge, pus, parasites, and calculi. However, demonstration of communication of such a dilated duct with a cyst, showing crumpled membranes, at or above the porta hepatis is highly suggestive of intrabiliary rupture of hydatid cyst and is the only direct sign of rupture.[3] MRI using MRCP with its multiplanar acquisitions enhances the demonstration of communication between the cyst and bile duct as shown by this case. Indirect signs of communicating rupture are based on evidence involving biliary tree and cystic contents and are reinforced by the demonstration of the direct sign.[1–3] A dilated biliary tree that contains hydatid material is an unequivocal sign of communicating rupture. Several changes in hydatid cyst contents secondary to rupture have been described, including irregularity of the wall, fat-fluid, and air-fluid levels.[2,3]

MRCP readily estabilishes the preoperative diagnosis of intrabiliary rupture of the hydatid cyst of liver, which is essential for its prompt management. ERCP may be employed for the removal of membranes from CBD and cyst drainage; this may be followed by operation for suturing the bile leakage without CBD exploration.[4] Primary surgical procedures include choledochotomy, evacuation and lavage of the CBD, closure of the CBD with T-tube drainage, and choledochoduodenostomy. External drainage may be performed with or without omentoplasty for the management of the cyst cavity.[4]
